# Assessment of anti-HIV-1 guide RNA efficacy in cells containing the viral target sequence, corresponding gRNA, and CRISPR/Cas9

**DOI:** 10.3389/fgeed.2023.1101483

**Published:** 2023-04-13

**Authors:** Alexander G. Allen, Cheng-Han Chung, Stephen D. Worrell, Glad Nwaozo, Rebekah Madrid, Anthony R. Mele, Will Dampier, Michael R. Nonnemacher, Brian Wigdahl

**Affiliations:** ^1^ Department of Microbiology and Immunology, Drexel University College of Medicine, Philadelphia, PA, United States; ^2^ Center for Molecular Virology and Gene Therapy, Institute for Molecular Medicine and Infectious Disease, Drexel University College of Medicine, Philadelphia, PA, United States; ^3^ Sidney Kimmel Cancer Center, Thomas Jefferson University, Philadelphia, PA, United States

**Keywords:** gene therapy, HIV-1 reservoir, CRISPR/Cas9, cure strategy, guide RNAs

## Abstract

The Clustered Regularly Interspaced Short Palindromic Repeats (CRISPR)/Cas9 gene editing system has been shown to be effective at inhibiting human immunodeficiency virus type 1 (HIV-1). Studies have not consistently used a trackable dual reporter system to determine what cells received the Cas9/gRNA to determine the overall knockdown of HIV. Some studies have used stably transduced cells under drug selection to accomplish this goal. Here a two-color system was used that allows tracking of viral protein expression and which cells received the CRISPR/Cas9 system. These experiments ensured that each gRNA used was a perfect match to the intended target to remove this variable. The data showed that gRNAs targeting the transactivation response element (TAR) region or other highly conserved regions of the HIV-1 genome were effective at stopping viral gene expression, with multiple assays demonstrating greater than 95 percent reduction. Conversely, gRNAs targeting conserved sites of the 5’ portion of the U3 region were largely ineffective, demonstrating that the location of edits in the long terminal repeat (LTR) matter with respect to function. In addition, it was observed that a gRNA targeting Tat was effective in a T-cell model of HIV-1 latency. Taken together, these studies demonstrated gRNAs designed to highly conserved functional regions have near 100% efficacy *in vitro* in cells known to have received the Cas9/gRNA pair.

## 1 Introduction

Human immunodeficiency virus type 1 (HIV-1) currently infects close to 37 million people around the world (Sengupta and Siliciano, 2018). At present, there are approximately 18.6 million people on anti-retroviral therapy (ART). Moreover, there are 1.8 million new infections every year. While ART is effective at limiting viral replication, it does not have the ability to cure a patient of HIV-1. This is in part due to the formation of the latent reservoir. During the HIV-1 life cycle, the genome of HIV-1 integrates into the host cells genome where it remains for the life of that cell (Sullivan et al., 2019). While most cells die within 24–48 h after infection there are a limited number of cells that become latently infected (Deeks, 2012) and remain transcriptionally silent for prolonged periods of time. The current estimated half-life for these cells is 44 months (Siliciano et al., 2003). Clinically, this has presented a problem if a patient did not maintain strict adherence to ART. Without constant therapy, HIV-1 will quickly rebound. Because of the aforementioned reasons a cure for HIV-1 has become an immediate public health priority.

There have been many different strategies used to attempt to cure HIV-1-infected cells. One of these strategies has been referred to as “shock and kill”. This strategy relies on a latency reversal agent (LRA) to induce viral transcription (Deeks, 2012). After the initiation of viral transcription, the shock and kill method can proceed in two distinct directions. In the first direction, upon the accumulation of viral proteins, the cell will die through the process of apoptosis. In the second direction, HIV-1-specific CD8^+^ T-cell recognize and destroy these cells as they present viral protein to be targeted by the immune response. Recent studies have shown that LRAs can induce viral transcription but the mechanisms that enhance direct killing of HIV-1-infected cells remain elusive (Huang et al., 2018). Another strategy aimed at eliminating HIV-1-infected cells has involved the use of the Clustered Regularly Interspaced Short Palindromic Repeats (CRISPR)/Cas9 gene-editing system (Dampier et al., 2014; Dampier et al., 2017; Dampier et al., 2018; Sullivan et al., 2019; Chung et al., 2020a; Atkins et al., 2021a). The CRISPR/Cas9 system works by having a guide RNA (gRNA), a 20 bp RNA oligomer, direct the Cas9 endonuclease to its target site. Once bound, Cas9 will induce a double-stranded break and in most cases this break will be repaired through non-homologous end joining (NHEJ). There have been a number of recent studies that have attempted to delete one of the HIV-1 co-receptors, CCR5 to engineer cells that are resistant to HIV-1 infection (Allen et al., 2018; Mohamed et al., 2021; Li et al., 2022). Another option that has involved the use of the CRISPR/Cas9 system has been to directly target the integrated provirus. Causing edits (insertion/deletions (InDels)) in the HIV-1 genome, especially in the LTR, may be enough to inactivate the promoter and silence LTR-directed HIV transcription. Another option has been used to design gRNAs that flank the integrated HIV-1 provirus and excise the entire provirus from the host genome (Yin et al., 2017). Previous studies have shown that excising the whole provirus was possible with gRNAs that target the long terminal repeats (LTR) (Hu et al., 2014). The HIV-1 LTRs are important components of the viral genome. The 5′ LTR primarily acts as a promoter for the virus while the 3’ LTR acts as a nascent poly-adenylation signal (Nonnemacher et al., 2004). The HIV-1 LTR is broken down into three main sections, U3, R, and U5. The U3 region harbors most of the transcription factor binding sites, while the R region encodes the secondary RNA structure known as the transactivation response element (TAR). TAR is vital for HIV-1 transcription to occur (Li et al., 2011; Dampier et al., 2014; Dampier et al., 2017; Dampier et al., 2018; Sullivan et al., 2019). When TAR has been transcribed, the viral accessory protein Tat then binds TAR and recruits the host positive transcription elongation factor (P-TEFb), which has been shown to be composed of Cyclin T1 and cyclin dependent kinase 9 (CDK9) (Kaminski et al., 2016a; Nguyen et al., 2017). The U5 region harbors a minimal amount of transcription factor binding sites.

Yet another hurdle to an HIV-1 cure has been the development of the viral quasispecies (Dampier et al., 2016). The efficacy of elected gRNAs with high coverage of intra- and inter-patient viral quasispecies has been required for the development of a generalizable anti-HIV-1 therapy using the CRISPR/Cas9 system (Roychoudhury et al., 2018; Sullivan et al., 2019). As HIV-1 replicates, mutations are incorporated into the viral genome, in part due to the error prone reverse transcriptase. The human restriction factor apolipoprotein B mRNA editing enzyme, catalytic subunit 3G (APOBEC3G) has also been shown to induce mutations in the HIV-1 genome further contributing to the expansion of viral quasispecies (Bruner et al., 2016). We have previously designed gRNAs using an extensive bioinformatic pipeline (Sullivan et al., 2019). This pipeline was designed to select gRNAs that recognize the high level of genomic heterogeneity within HIV-1, while still targeting important functional regions and minimizing the potential for off-target events. Moreover, these studies have shown that gRNAs designed with our pipeline for targeting the HIV-1 LTR performed better than previously published LTR gRNAs (Wang et al., 2018; Campbell et al., 2019). While these gRNAs performed better, HIV-1 knockdown still did not reach 100 percent, leading to the question of why, with delivery being the primary variable. Here we have utilized a dual fluorescent system to track Cas9-treated cells and HIV-1-infected cells to determine the impact of CRISPR/Cas9 effectiveness in cells that are known to have received and expressed the gRNA/Cas9 package (i.e., known to have received the CRISPR/gRNA-based therapeutic). This study demonstrated that the pipelines top hit, selected molecular gRNA target 1 (SMRT1), was the most effective at reducing LTR-mediated transcription from the gRNAs tested.

## 2 Materials and methods

### 2.1 Cell lines and culture conditions

Human embryonic kidney (HEK)-293T/17 cells were purchased from American Type Culture Collection (ATCC). TZM-bl cells were obtained from the NIH AIDS Reagent Bank, along with the J-Lat 10.6 cell line. HEK-293T/17 and TZM-bl cells were cultured in DMEM with 10% heat-inactivated FBS (v/v) with 100 mg/ml of penicillin and 100 μg/ml of streptomycin. J-Lat 10.6 cells were cultured in RPMI-1640 with 10% heat-inactivated fetal bovine serum (FBS) with 100 mg/ml of penicillin and 100 μg/ml of streptomycin.

### 2.2 Cloning of gRNAs and NL4-3∆Env acquisition

The lentiviral (LV) backbone was purchased from Addgene (57826). Forward and reverse oligonucleotides were ordered from Integrated DNA Technologies (IDT). gRNAs were cloned into this backbone as previously described (Chung et al., 2020b). Since the gRNAs SMRT 1 and 4 had the potential to target the LV recombinant plasmid backbone, the LV backbone sequence was examined. With respect to the SMRT1 gRNA construct, the backbone LV plasmid contained the target and required PAM sequence in both the 5′ and 3′LTR regions. To ensure no feedback was encountered from the gRNA, site-directed mutagenesis was used to mutate the PAM for this gRNA at positions 288 to 290 in the 5′LTR and 10,354 to 10,357 in the 3′ LTR from GGG to TAA, respectively. This was confirmed *via* Sanger sequencing. This eliminated the negative feedback issue as confirmed by RFP expression (see Supplementary Figure S1, Supplementary Figure S2). The SMRT4 gRNA did not have a PAM sequence present at the target sites. The sequence at position 273 to 275 in the 5′LTR and 10,340 to 10,343 in the 3′LTR was TCG, so no mutation was required. The NL4-3∆Env molecular clone was acquired from the NIH AIDS reagent bank (catalog number: 11100).

### 2.3 Transfection of HEK-293T cells

Lipofectamine 3,000 was used as described by the manufacturer (Invitrogen). HEK-293T cells were seeded in a 12-well plate at 150,000 cells per well. Twenty four hours post seeding, cells were transfected with 1 µg of plasmid Cas9/gRNA DNA. Twenty four hours post Cas9/gRNA transfection, the molecular clone NL4-3∆Env (1 μg) was transfected into HEK-293T cells. For experiments where NL4-3∆Env was transfected before Cas9/gRNA transfection, the same cell number and amount of DNA was used. For experiments when Cas9/gRNA and NL4-3∆Env were used simultaneously, 60,000 cells were seeded in a 24-well plate. Twenty four hours post seeding, 750 ng of NL4-3∆Env was used, and 250 ng of Cas9/gRNA plasmid was used. In all experiments, flow cytometry and fluorescent microscopy was performed 48 h after the last transfection.

### 2.4 Flow cytometry for HEK-293T cells

Forty eight hours after the last transfection, cells were treated with trypson and lifted cells were placed into FACS tubes with 1 ml of warmed DMEM. All spin steps were performed at 500xg for 3 min. Cells were then washed twice with FACS wash buffer, 96% PBS, 3% heat-inactivated FBS, and 1% 1M HEPES. Cells were then fixed with 1% paraformaldehyde for 15 min. After fixation, cells were subjected to centrifugation and resuspended in FACS wash buffer. All flow samples were run on a BD LSR Foretssa, with 50,000–100,000 events collected.

### 2.5 Fluorescent microscopy

Forty eight hours after the last transfection, cells were imaged using an Olympus X81 microscope. All images were taken at a ×10 magnification. The image is representative of the well.

### 2.6 Generation of NL4-3∆Env pseudotype virus

HEK-293T cells were seeded at 2.5 × 10^6^ cells in a 100 mM dish. Twenty four hours post seeding, cells were transfected using the CaPO4 transfection method. Briefly, 1X HEPES buffered saline (HBS) was made by adding 5 g HEPES (Acid), 8 g NaCl, 1 g Dextrose, 3.7g KCl, and 10 ml of Na2HPO4(7H2O) for a 1 L solution. CaCl_2_ (2.5 M) was made by adding 36 g of CaCl_2_ anhydrous to 100 ml of water. HBS (1X, 500 µl) was added to a microcentrifuge tube. VSV-G envelope plasmid (pMD2.G; 10 µg) and NL4-3∆Env (10 µg) plasmid were added and co-transfected to 293T cells. After addition of both plasmids, 30 µl of the stock CaCl_2_ (2.5 M) was added and the entire solution was mixed and incubated for 20 min at room temperature. After the incubation step, the entire solution was added drop-wise to a 100 mM dish. Twelve hours post transfection, the media was changed and supernatants were collected at 24 and 48 h post media change. Supernatants from 24 to 48 h were pooled and clarified by spinning the supernatant at 1000xg for 5 min. After clarification, supernatant was put through a 0.45 µm filter. To concentrate the virus the Lenti-X concentrator solution was used as described by manufacturer (Takara).

### 2.7 Construction of LV vectors for Cas9/gRNAs

LV vectors were constructed in a manner similar to how the pseudotype NL4-3∆Env virus was constructed with minimal changes. Briefly, LV was made by using the same CaPO4 transfection method. Eight µg of a VSV-G plasmid was used with 12 µg of the packaging plasmid psPAX2 and 20 µg of the Cas9/gRNA transfer plasmid. Media change and supernatant collection times were the same as described previously (Chung et al., 2020b).

### 2.8 Fluorescent titer of virus production

A fluorescent titter protocol was adopted from Addgene (https://www.addgene.org/protocols/fluorescence-titering-assay/). Briefly, HEK-293T cells were seeded in a 6-well plate at 75,000 cells per well. Twenty four hours post seeding, 10-fold serial dilutions of virus were made for each Cas9/gRNA LV and the NL4-3∆Env virus. Cell media was aspirated and 1 ml of virus-containing media was added dropwise to the cells. Twenty four hours after transduction, media was changed, and cells were allowed to grow for another 24 h. Forty eight hours after transduction, cells were trypsonized and run through the flow cytometry protocol described above.

### 2.9 Infection of HEK-293T cells with pseudotype NL4-3∆Env virus

HEK-293T cells were seeded in 12-well plates at 150,000 cells/well. Twenty four hours later cells were transfected with the Cas9/gRNA plasmid using lipofectamine 3,000 as described by the manufacturer (ThermoFisher Scientific). Twenty four hours post transfection, cells were infected with pseudotyped NL4-3∆Env virus at an MOI of 0.3. Cells were allowed to rest for 48 h. After this rest period, cells were processed for flow cytometry.

### 2.10 Beta-galactosidase expression analysis

TZM-bl cells have an integrated LTR that drives beta-galactosidase (Wei et al., 2002). Cells were seeded at 20,000 cells per well in a 96-well flat bottom plate. Twenty four hours post seeding, cells were transfected with a 10:1 ratio of Cas9/gRNA plasmid to NL4-3∆Env plasmid using Lipofectamine 3,000, as described by the manufacturer (ThermoFisher Scientific). A total of 100 ng of plasmid DNA was used. Forty eight hours post transfection, cells were processed for beta-galactosidase expression using the Galacto-Star™ One-Step beta-Galactosidase Reporter Gene Assay System as described by the manufacturer (ThermoFisher Scientific). Briefly, cell culture supernatant was aspirated and the supplied lysis buffer (30 µl per well) was added to cell monolayers. Lysis buffer was allowed to sit on cells for 10 min on an orbital shaker. During that time, substrate was added to the supplied diluent at a 1:50 dilution factor (100 µl of substrate/diluent solution per well). Ten µl of cell lysate was added to the 100 µl of substrate/diluent solution. Cells were then incubated at room temperature in the dark for 60 min. After the 60-min incubation, chemiluminescence was read on a GlowMax 96 dual injector plate reader.

### 2.11 LV transduction and stimulation of J-Lat 10.6 cells

J-Lat 10.6 cells were seeded in a 96-well v-bottom plate at 25,000 cells per well in 100 µl. Twenty four hours post seeding cells were transduced with LV. An MOI of 1 was used for each LV. An MOI of 2 was used for combination treatments (MOI 1 for each virus). Polybrene (8 μg/ml) was added to LV before addition of LV to cells. Cells were then spinoculated using the following parameters; 1000xg for 60 min at 30 °C. After the spinoculation, cells were incubated as a pellet overnight. After the overnight incubation cells were transferred to a 24-well plate. Forty eight hours post-transduction cells were checked for RFP expression by fluorescent microscopy. On the same day, cells were split in half and seeded into a 12-well plate. Forty eight hours later cells were FBS spiked with 1 ml of media. Twenty four hours later, cells were spun down and resuspended in fresh media. Twenty four hours before running flow cytometry, J-Lats were stimulated with either Phorbol 12-myristate 13-acetate (PMA) and Ionomycin (Sigma) or tumor necrosis factor alpha (TNF-α). PMA was used at 50 ng/ml and Ionomycin was used at a 1 µM concentration. TNF-α was used at a concentration of 20 ng/ml. PMA + Ionomycin was chosen over CD3/CD28 beads because J-Lat cells downregulate CD3, rendering the beads ineffective stimulating agents (Spina et al., 2013). Twenty four hours post stimulation, cells were processed for flow cytometry. Cells were gated on single, live RFP + cells with the gating strategy as previously described (Chung et al., 2020b).

### 2.12 Correlations between both diversity and predicted CRISR activity to observed CRISPR activity

HIV-1 subtype B sequences (424,893 in total) were retrieved from the Los Alamos National Laboratory (LANL) HIV sequence database. The sequence diversity in this study was determined by a summation of Shannon entropy over the 20-bp window. The sequence diversity was defined as:

$$-\sum_{i=1}^{20}\underset{j}{\sum }p_{i,j}\times \log_{2}p_{i,j}$$

where i = nucleotide position from 1 to 20 within the 20-bp window; j = nucleotide identity, [A, C, G, T]; p = nucleotide probability (e.g., p_10,A_ represent the probability of A at 10th bp in a given 20-bp window). The range of the sequence diversity is [0,40]. A window with 40 means every base at every position was random. A diversity of 0 means every sequence in this window has converged to one variant. The predicted CRISPR activity was the percentage of fully overlapped HIV-1 sequence that possessed a cutting frequency determination (CFD) score of 0.569 or above against the comparison gRNAs. A predicted activity of 0.5 means there were 50% of HIV-1 sequence variants in the LANL database that had CFD score over 0.569. The number of tested sequences for each gRNA varied due to presence of many partial HIV-1 genome sequences in the LANL database. The Spearmen correlation coefficient was conducted and a two-tailed *t*-test was used to test the significance of correlation (*p < 0.05).

## 3 Results

### 3.1 Selection of gRNAs used and their sequence similarity to NL4-3 and R7

**FIGURE 1:**
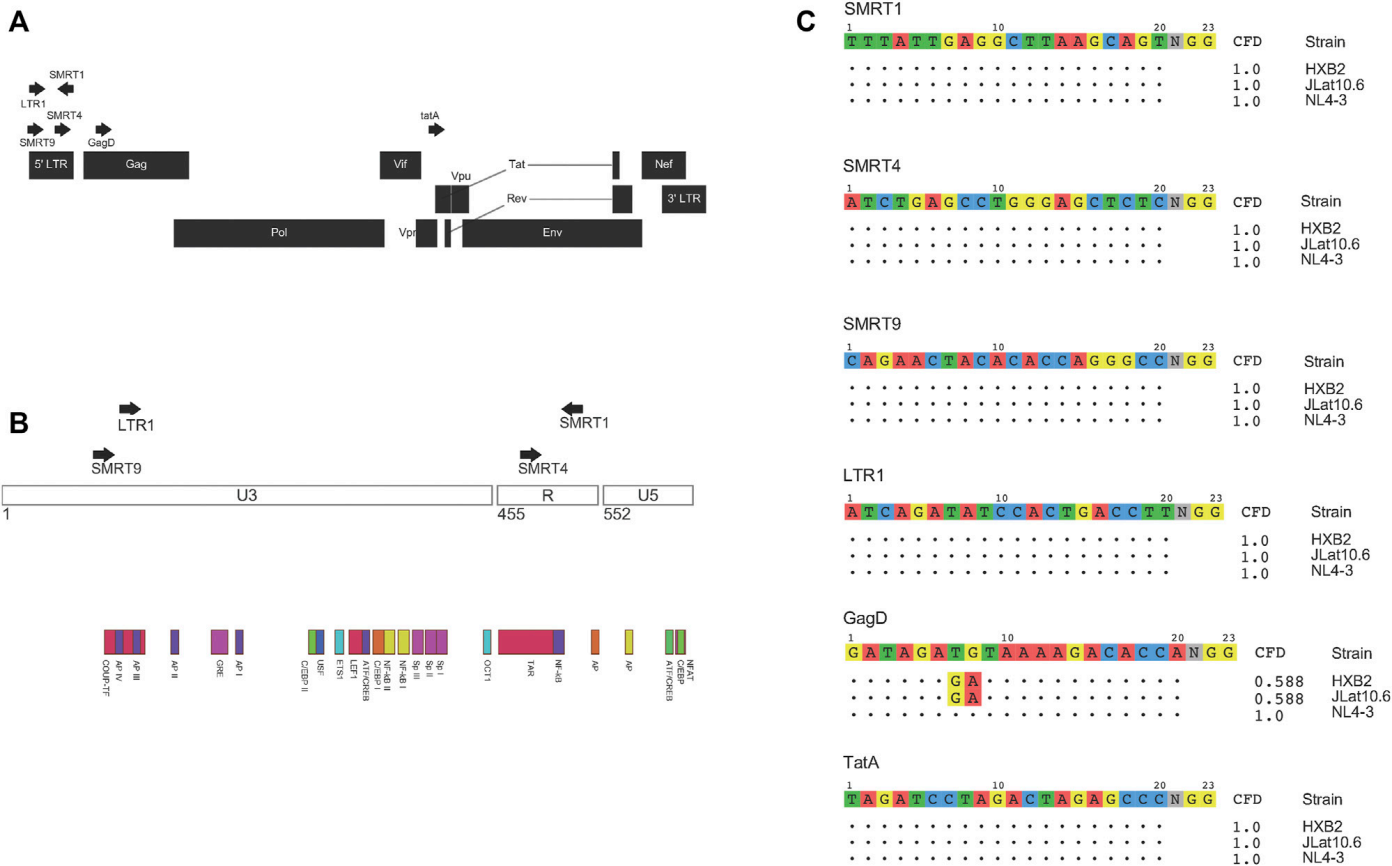
gRNAs used and their match to NL4-3, R7, and HXB2 HIV-1 strains. Schematic representation of where the gRNAs used in this study targeted the HIV-1 genome **(A)**. Magnified view of the HIV-1 LTR and where different gRNAs target inside the LTR **(B)**. All gRNAs were then aligned to different strains of HIV-1. Both NL4-3 and R7 were used in this study, while HXB2 is a commonly referred to HIV-1 genome. Based on where the mismatches were between the gRNA and target sequence a CFD score was calculated based on a previously published penalty matrix. CFD scores range from 0 to 1, 0 being no predicted binding and 1 being a high probability of binding **(C)**.

In this study, four LTR targeting gRNAs and two gRNAs that targeted different regions of the genome, one in Gag (Yin et al., 2017) and one in Tat (Ophinni et al., 2018) (Figure 1A) were used. A mixture of previously published LTR targeting gRNAs (SMRT4 and LTR1) and gRNAs that were top ranked from our bioinformatic pipeline (SMRT1, SMRT4, and SMRT9) were used (Kaminski et al., 2016b; Sullivan et al., 2019). SMRT4 was identified independently in our pipeline and another recent report (Mefferd et al., 2018). For the gRNAs targeting the LTR, gRNAs targeted the U3 region (LTR1 (14) and SMRT9) and the TAR region (SMRT1 and SMRT4) (Figure 1B). One of the first questions to be addressed experimentally was to determine if gRNAs were delivered to cells would there be a high probability of binding and cleaving their targets subsequent to delivery. To accomplish this task, the percent match to the HIV-1 NL4-3 strain and to the HIV-1 R7 strain contained within the J-Lat 10.6 cell line used in this study was calculated. All gRNAs used in these analyses had a 100% match to NL4-3 except the previously published gRNA designated GagD which had two mismatches at the 7,8 position of the gRNA (Kaminski et al., 2016c). Even though there were mismatches this gRNA was included due to it having been analyzed in multiple studies to date and included in the current clinical trial EBT-101 (ClinicalTrials.gov Identifier: NCT05144386). Based on analysis using the CFD matrix, these mismatches predict a 42 percent reduction in binding and cleavage (Figure 1C). Moreover, the gRNAs were compared to a common target HIV-1 reference genome known as HXB2. All gRNAs except GagD were a perfect match for HXB2 (Figure 1C).

### 3.2 gRNAs targeting the TAR region of the LTR yielded the greatest reduction in HIV-1 gene expression

**FIGURE 2:**
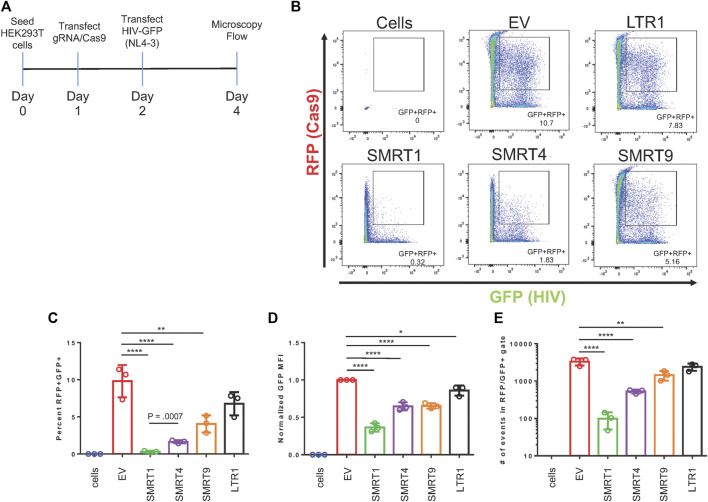
gRNAs targeting TAR have the greatest effect on LTR-mediated HIV-1 gene expression. HEK-293T cells were transfected with the all in one Cas9 vector system that also expressed the gRNA and RFP. Twenty four hours post transfection, an NL4-3 GFP molecular clone, referred to as HIV-1 GFP was transfected into the same cells. Forty eight hours after transfection with Cas9, cells were processed for flow cytometry. **(A)** A schematic representation of the experimental timeline. **(B)** Representative FACS plots of detecting double-positive cell populations (RFP + GFP+). Double-positive events are shown in the gated box. EV = empty vector, expresses Cas9 and RFP with no gRNA. Cells are HEK-293T cells that were untreated with any plasmid. **(C)** Quantitative analysis of gated cell populations (GFP + RFP+), **(D)** Mean fluorescent intensity (MFI) of GFP from double-positive gated cells, **(E)** total number of events in the double-positive gate. Error bars represent ± SD. Statistical significance was determined by using a One-way ANOVA with multiple comparisons. *p* = * <.05, ** <.01, *** <.001, **** <.0001. Data was collected from three independent experiments. 50,000 events were collected from each treatment group.

The selected gRNAs were cloned into a lentiviral vector (Addgene: 57,826) that contains a human U6 Pol III promoter for the gRNA and a SFFV (spleen focus forming virus) promoter for Cas9 expression. In addition to the Cas9 protein, the SFFV promoter also drove a red fluorescent protein (RFP) as an indicator of Cas9 expression using live cell imaging. This versatile lentiviral vector could elicit functional CRISPR/Cas9 delivery by direct transfection or lentiviral production for stable transduction. HIV-1 gene expression was measured using an HIV-1 NL4-3 Env-GFP plasmid (HIV-1 GFP). This clone was based off an NL4-3 backbone but has eGFP inserted into the *env* gene (Zhang et al., 2004). Assessment of the GFP signal was used as a measure of HIV-1 gene expression. HEK-293T cells were transfected with the plasmid encoding the gRNA and Cas9, or an empty vector (EV). The EV plasmid does not express a gRNA but does express Cas9 and RFP. Twenty four hours after gRNA/Cas9 transfection, cells were transfected again with the HIV-1 GFP construct. An experimental timeline is illustrated in Figure 2A. Forty eight hours post HIV-1 GFP transfection, the cells were processed for flow cytometry and fluorescent micrographs were taken (Figure 2B and Supplementary Figure S1). By utilizing a dual fluorescent system, cells expressing GFP + RFP- (cells that did not receive Cas9 but received HIV-1 GFP) and cells expressing GFP + RFP+ (cells that received both Cas9 and HIV-1 GFP) were able to be distinguished. To determine the effect of Cas9 treatment, GFP + RFP + populations were gated from live single cells. EV was used as a negative control. SMRT1 and SMRT4 yielded the greatest reductions in percentage of cells that were GFP + RFP+ (97.0% and 83.3%, respectively), while the reductions from SMRT9 and LTR1 were modest (58.8% and 31.1%, respectively) (Figure 2C). The cells were further analyzed for the mean fluorescent intensity (MFI) of the GFP + cells that were in the double-positive gate. The SMRT1 gRNA showed the greatest reduction in GFP MFI (63.6% reduction). This was significantly greater than the reduction in SMRT4-treated cells (35.2% reduction; Figure 2D). Moreover, the total number of events in the double-positive gate were examined and showed that SMRT1 and SMRT4 gave the greatest reductions in the total number of events with SMRT1 showing significantly more reduction than SMRT4 (*p* = .0007; Figure 2C). While SMRT1 and SMRT4 targets are only 20 nucleotides apart, they bind to two separate parts of the TAR secondary RNA structure; SMRT4 binds closer to the loop where the viral protein Tat interacts. SMRT1 binds at the base of the TAR secondary structure. These cells were also imaged with a fluorescent microscope. Images were taken by combining bright field (BF), TRITC (RFP), and FITC (GFP) in order to visualize the double-positive populations. In concordance with the flow cytometry data, the highest number of double-positive cells came from the SMRT9-and LTR1-treated cells, while the lowest number of double-positive cells came from the SMRT1-and SMRT4-treated cells (Supplementary Figure S1). Taken together, these data demonstrated that when SMRT1 was delivered to cells it significantly reduced LTR-driven gene expression. Moreover, this data demonstrated that even when two gRNAs are targeting the same region of the HIV-1 LTR there can be significant differences in terms of knockdown potential.

### 3.3 gRNAs targeting TAR were able to reduce LTR-mediated gene expression in a post exposure setting

**FIGURE 3:**
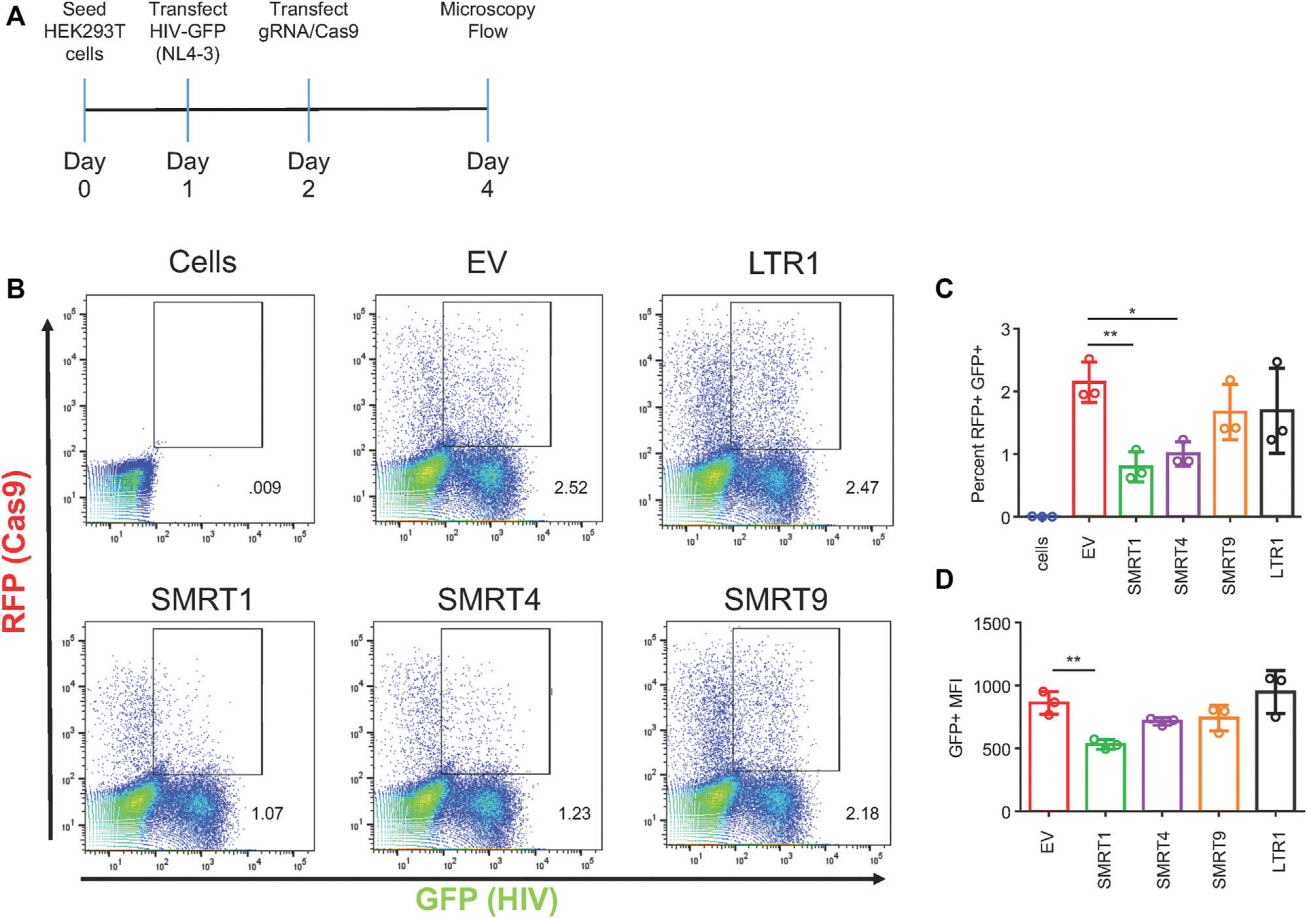
Only gRNAs targeting TAR are able to significantly reduce viral gene expression in a post-exposure setting. HEK-293T cells were transfected with HIV-1 GFP plasmid for 18 h. After the 18-h incubation, cells were transfected with the all in one Cas9 plasmid. Forty eight hours after transfection with Cas9, cells were processed for flow cytometry. **(A)** A schematic of the experimental timeline. **(B)** Representative FACS plots showing gating for double-positive cells. **(C)** Quantitative representation of double-positive cells, **(D)** Mean fluorescent intensity of GFP from double-positive cells. Error bars represent ± SD. Statistical significance was determined by using a One-way ANOVA with multiple comparisons. *p* = * <.05, ** <.01. Data was collected from three independent experiments. Fifty thousand events were collected from each treatment group.

The next set of experiments were designed to determine the effect of the CRISPR/Cas9 system in cells that were already expressing the HIV-1 GFP construct. In this experiment, HIV-1 GFP was transfected into HEK-293T cells 18 h before transfecting the gRNA constructs (Figure 3A). The 18-h time point was selected to mitigate the amount of cell death from HIV-1 GFP. Only the gRNAs targeting TAR (SMRT1, SMRT4) were able to decrease the amount of GFP observed (62.9 and 53.3 percent reduction, respectively) (Figure 3C). In addition, the MFI of GFP was examined from the double-positive gate. It showed that the SMRT1-treated cells demonstrated a significant reduction in GFP MFI, while all other gRNAs did not yield a significant reduction (Figure 3D). This indicated that once viral transcription was initiated, there may be cellular proteins (transcription factors) that block the CRISPR system. Moreover, these results indicated that targeting upstream in the LTR after transcription has been initiated is ineffective at stopping viral transcription.

### 3.4 HIV-1 expression is diminished when CRISPR/cas9 was delivered at reduced concentrations

**FIGURE 4:**
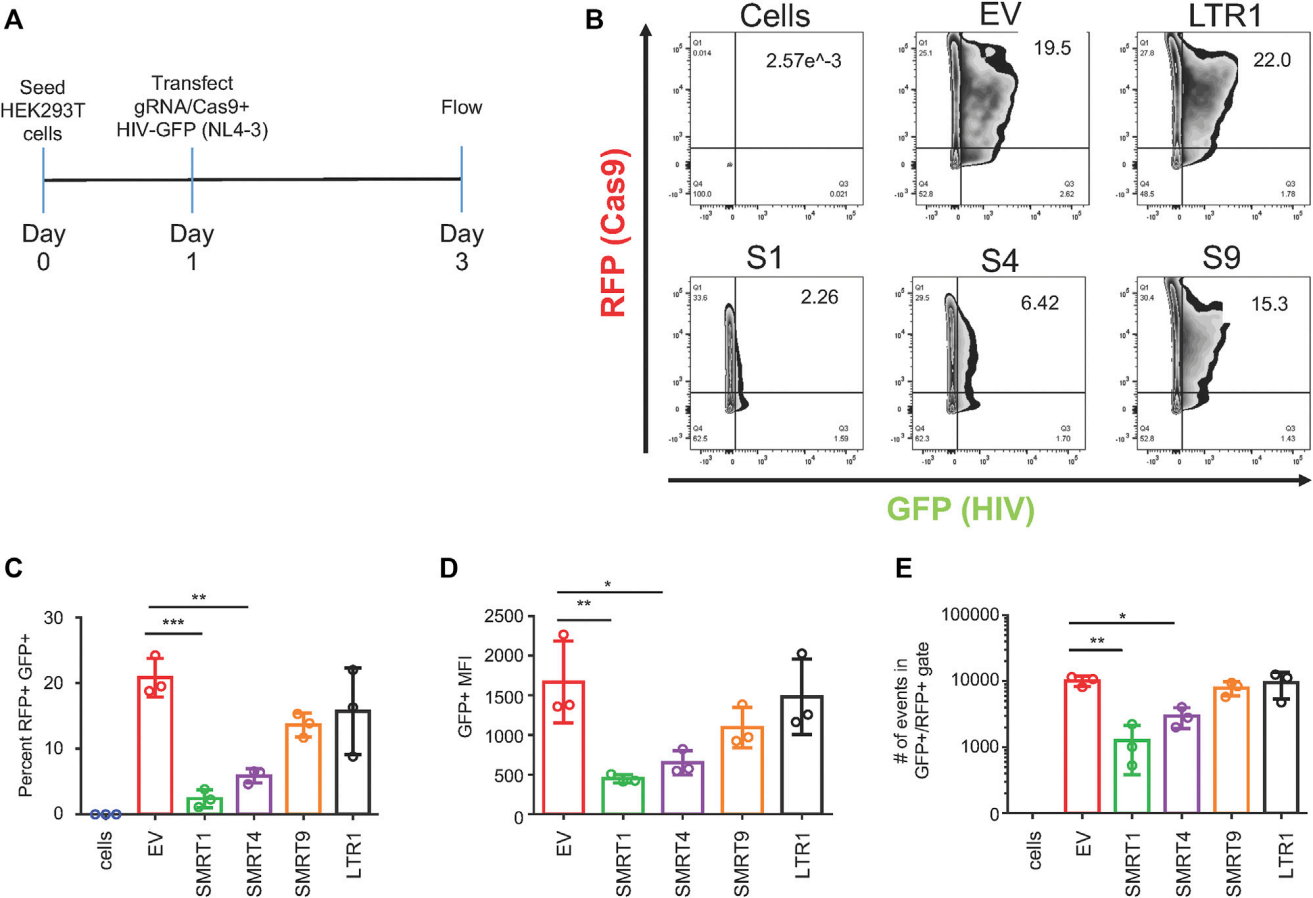
Co-transfections with a 3:1 ratio of HIV-1 GFP to Cas9 maintains SMRT1 and SMRT4s ability to significantly reduce HIV-1 GFP expression. HEK-293T cells were transfected in a 24-well plate with 750 ng of HIV-1 GFP and 250 ng of Cas9 plasmid. Forty eight hours post transfection, cells were processed for flow cytometry. **(A)** A schematic representation of the experimental timeline. **(B)** Representative FACS plots showing gating for double-positive cells. **(C)** Quantitative representation of double-positive cells, **(D)** Mean fluorescent intensity of GFP from double-positive cells, **(E)** total number of events in the double-positive gate. Error bars represent ± SD. Statistical significance was determined by using a One-way ANOVA with multiple comparisons. *p* = * <.05, ** <.01, *** <.001. Data was collected from three independent experiments.

The next set of experiments were then designed to address how the ratio of the CRISPR/Cas9 system to HIV-1 would affect the results with a 3:1 ratio of HIV-1 GFP to the gRNA constructs (750 ng HIV-1 GFP, 250 ng gRNA) being used to optimize the level of inhibition achieved with each gRNA. To maximize the likelihood that these two plasmids would interact in the same cell, a co-transfection system was utilized. After co-transfection, cells were allowed to incubate for 48 h, after which time cells were processed for flow cytometry and fluorescent microscopy (Figure 4A and Supplementary Figure S2, respectively). Similar to the results in Figure 2, gRNAs targeting TAR were the most effective at decreasing GFP expression. SMRT1 and SMRT4 yielded 88.48 and 71.92 percent reductions, respectively, while SMRT9 and LTR1 yielded 34.57 and 24.63 percent reductions, respectively (Figures 4B,C). Upon analysis of the GFP MFI, both the TAR-targeting gRNAs yielded significant reductions, while SMRT9 and LTR1 did not yield significant reductions in GFP MFI (Figure 4D). Moreover, significant reductions in the number of events in the double-positive gate with SMRT1 and SMRT4, but not with SMRT9 and LTR1 were observed. Fluorescent microscopy was also used to qualitatively measure the number of double-positive cells after co-transfection. The fluorescent microscopy clearly demonstrated a lack of double-positive cells in the SMRT1-and SMRT4-treated cells (Supplementary Figure S2). Taken together, these results indicated that even when adding an increased amount of HIV-1 GFP DNA the CRISPR Cas9 system was able to significantly decrease the amount of LTR-mediated gene expression.

### 3.5 CRISPR system ablates HIV-1 GFP expression in a pseudovirus infection system

**FIGURE 5:**
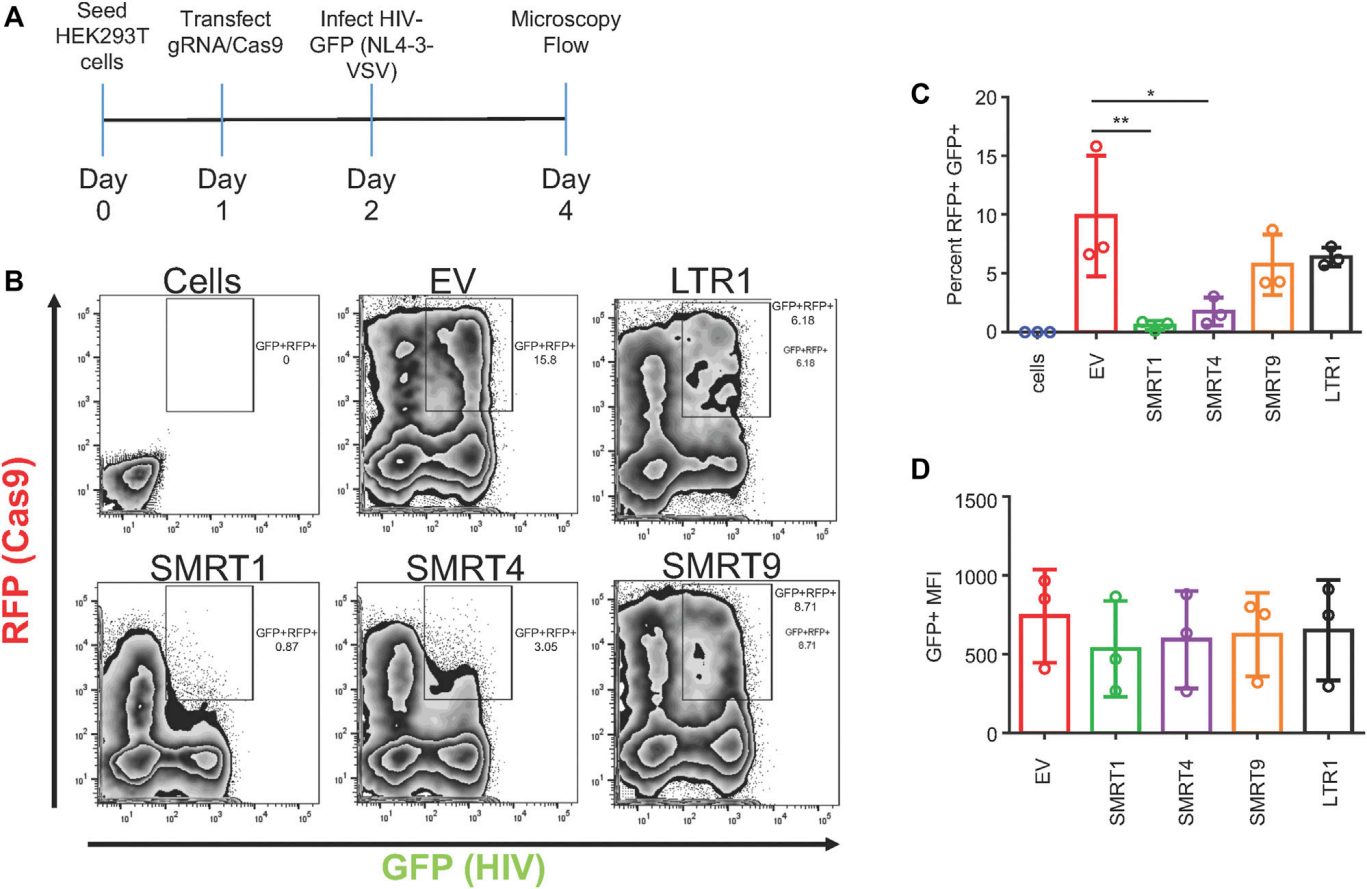
gRNAs targeting TAR were able to suppress HIV-1 GFP expression in a pseudotype infection. HEK-293T cells were transfected with the all in one Cas9 plasmid. Twenty four hours post transfection, cells were infected at an MOI of 0.3 with VSV-G pseudotyped HIV-1 GFP. Forty eight hours post-infection, cells were processed for flow cytometry. **(A)** A schematic representation of the experimental timeline. **(B)** Representative FACS plots showing gating for double-positive cells. **(C)** Quantitative representation of double-positive cells. **(D)** Mean fluorescent intensity of GFP from double-positive cells. Error bars represent ± SD. Statistical significance was determined by using a One-way ANOVA with multiple comparisons. *p* = * <.05, ** <.01. Data was collected from three independent experiments.

While plasmids represent a very useful tool for transient expression studies, they are limited in studies of retrovirus gene expression in that they do not integrate into the host genome. To circumvent this limitation, a pseudotype virus system was utilized as an alternate infection strategy. The gRNA constructs were transfected into HEK-293T cells 24 h before infection (Figure 5A). Infections were performed at a multiplicity of infection (MOI) of 0.3. Forty eight hours after infection, the cells were processed for flow cytometry. Similar to what has been shown previously, SMRT1 and SMRT4 significantly decreased the amount of GFP + RFP + double-positive cells when compared to EV (94.13 and 82.3 percent reduction, respectively; Figure 5B). SMRT9 and LTR1 yielded modest reductions in GFP + RFP + double-positive cells (41.9 and 35.4 percent reduction, respectively), both of which were not significant (Figure 5C). Furthermore, relative reductions in GFP mean fluorescent intensity (MFI) among the gRNAs examined were similar to what was observed in Figure 2, (Figure 5D). This data demonstrated that with integrated provirus the gRNAs were still able to cleave the LTR and disrupt LTR-mediated transcription at a high level (Figure 5).

### 3.6 Combination gRNAs lead to a significant reduction in LTR-driven reporter gene expression in TZM-bl cells

**FIGURE 6:**
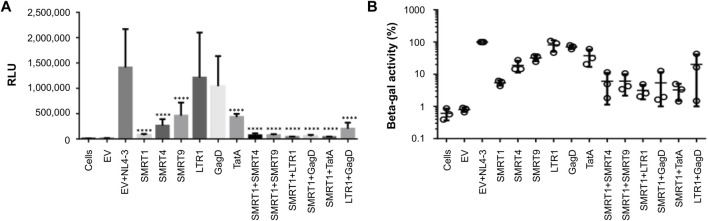
Combination gRNAs lead to a near 100 percent reduction in LTR-mediated reporter gene expression. TZM-bl cells were seeded in 96-well plates at 20,000 cells per well. Cells were then co-transfected with HIV-1 GFP and Cas9 plasmids. Forty eight hours post-transfection cells were processed for beta-galactosidase expression. **(A)** Quantitative representation of relative light units (RLU) with different gRNA treatment groups. **(B)** The EV with NL4-3 treated group was normalized to 100 percent, different gRNAs in single and combination treatments were compared to the EV + NL4-3 group. Error bars represent ± SD. Statistical significance was determined by using a One-way ANOVA with multiple comparisons. *p* = **** <.0001. Data were collected from three independent experiments, each performed in technical quadruplicate.

Recent reports have shown that combinations of gRNAs have been able to synergize and further reduce HIV-1 gene expression (Wang et al., 2016; Darcis et al., 2019). In addition, it has been shown that gRNAs targeting regions of the HIV-1 genome that are separated by a greater distance on the viral genome are more effective in knock down studies performed *in vitro* (Wang et al., 2016). Given this observation, the lead candidate gRNA, SMRT1, was used and independently multiplexed with five separate gRNAs targeting the LTR, Gag, and Tat. In this *in vitro* experimental system, TZM-bl cells containing an integrated LTR capable of driving beta-galactosidase production in response to the viral accessory protein Tat was used (Wei et al., 2002). In this case, Tat was provided by the HIV-1 GFP plasmid. Previous reports have used differing ratios of Cas9/gRNA plasmid to molecular clone to achieve the greatest reduction in HIV-1 gene expression (Yin et al., 2017; Mefferd et al., 2018). Therefore, an experiment measuring levels of knockdown achieved with differing ratios of Cas9/gRNA:HIV-1 GFP plasmid was performed. The results of this analysis demonstrated that a 10:1 ratio of Cas9/gRNA:HIV-1 GFP plasmid was significantly decreased with SMRT1 (Supplementary Figure S3). This ratio was then used in the same TZM-bl system to examine the efficiency of the gRNAs individually and in combination. Cells treated with LTR1 or GagD gRNA alone did not yield a significant reduction in LTR-driven gene expression. However, when used in combination, LTR1 and GagD produced a significant reduction in LTR-driven gene expression, recapitulating what has been previously shown by Khalili and coworkers (Kaminski et al., 2016b; Bella et al., 2018; Dash et al., 2019; Mancuso et al., 2020). All other gRNAs singly or in combination led to a significant reduction in LTR-driven gene expression (Figures 6A,B). SMRT1-treated cells demonstrated a 94.6 percent reduction, while the combined SMRT1 and TatA gRNA treatment led to a 96.8 percent reduction (within the error of the assay, this result could be interpreted as near or complete inhibition). Interestingly when SMRT1 was combined with other gRNAs, this did not lead to an additional reduction in LTR-driven gene expression. This is likely due to the fact that SMRT1 alone was able to reduce LTR-mediated gene expression almost down to basal levels, indistinguishable from background levels.

### 3.7 Combination gRNAs lead to a significant reduction in HIV-1 reactivation from latency in provirus-containing J-Lat 10.6 cells

**FIGURE 7:**
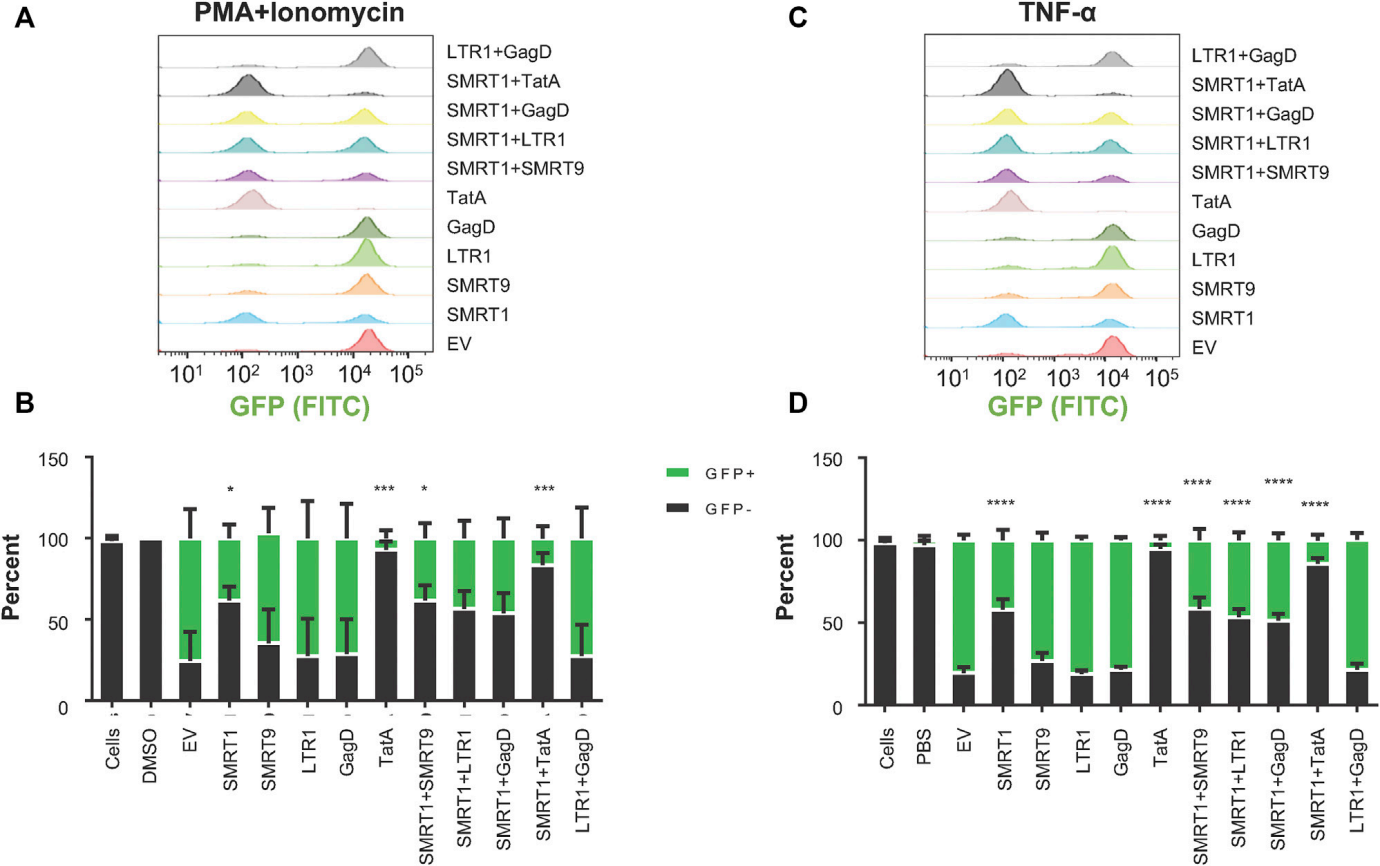
gRNAs SMRT1 and TatA were the most effective at stopping latency reversal in a T-cell model of HIV-1 latency. J-Lat 10.6 cells were seeded at 25,000 cells per well in a 96-well v-bottom plate. Twenty four hours post-seeding, cells were spinoculated with LV containing different gRNAs at an MOI of 1. Post-spinoculation, cells were allowed to incubate overnight. Cells were allowed to rest for 5 days before being stimulated with either PMA + Ionomycin (50 ng/ml, 1 µM) or TNF-α (20 ng/ml) for 24 h. Twenty four hours after stimulation cells were processed for flow cytometry. (**A, C**) Histograms were used to visualize populations that were GFP + or GFP-. (**B, D**) Grouped bar graphs represent cells that are GFP+ (green) and GFP- (black). Error bars represent ± SD. Statistical significance was determined by using a One-way ANOVA with multiple comparisons. *p* = * <0.05, *** <0.001, **** <0.0001. Data were collected from three independent experiments.

It has been previously demonstrated that for most individuals on ART, HIV-1 infection is effectively suppressed with latent provirus residing primarily in the resting memory T-cell population. Given this observation, the latently infected Jurkat cell line known as J-Lat (Jordan et al., 2003) was used as an initial T-cell model of latent infection to examine the targeting efficacies of anti-HIV-1 gRNAs. These cells have one near full-length provirus copy of the R7 strain of HIV-1. In place of *nef* in this T-cell line, GFP has been inserted and there is a frameshift mutation in the *env* gene rendering the virus replication incompetent. Under basal conditions, there was minimal GFP expressed indicating that there was very little, if any, viral gene expression in this cell line suggesting the establishment of a latent infection. Unlike other cell lines with integrated HIV-1 proviruses, the J-Lat cell line does not have mutations in the TAR or Tat regions (Emiliani et al., 1996; Emiliani et al., 1998). In order to deliver the Cas9/gRNAs to these cells, LV vectors were generated. Significant increases in the number of cells that remained GFP- (no viral protein production) were observed with SMRT1, TatA, SMRT1+SMRT9, and SMRT1+TatA, when stimulated with PMA + Ionomycin (Figure 7A). When J-Lats were stimulated with TNF-α, significant increases in the GFP-population were found with SMRT1, TatA, SMRT1+SMRT9, SMRT1+LTR1, SMRT1+GagD, and SMRT1+TatA (Figure 7C). The most effective gRNA alone was TatA, yielding 92.9 percent GFP- in PMA + Ionomycin-treated cells and yielding 94.8 percent GFP-negative in TNF-α-treated cells (Figure 7 B, D). This is in congruence with a previously published report that utilized TatA (Ophinni et al., 2018). The most effective combination was SMRT1+TatA. In SMRT1+TatA treated and PMA + Ionomycin stimulated cells, 83.6 percent were GFP-, while in TNF-α-treated cells this number was 85.7. These data taken together has indicated that while SMRT1 was still effective at stopping HIV-1 transcription, it was not as effective as TatA. Moreover, these data have also demonstrated that using different stimulation conditions yielded a similar outcome.

### 3.8 Positive correlations between diversity, predicted activity, and observed activity

**FIGURE 8:**
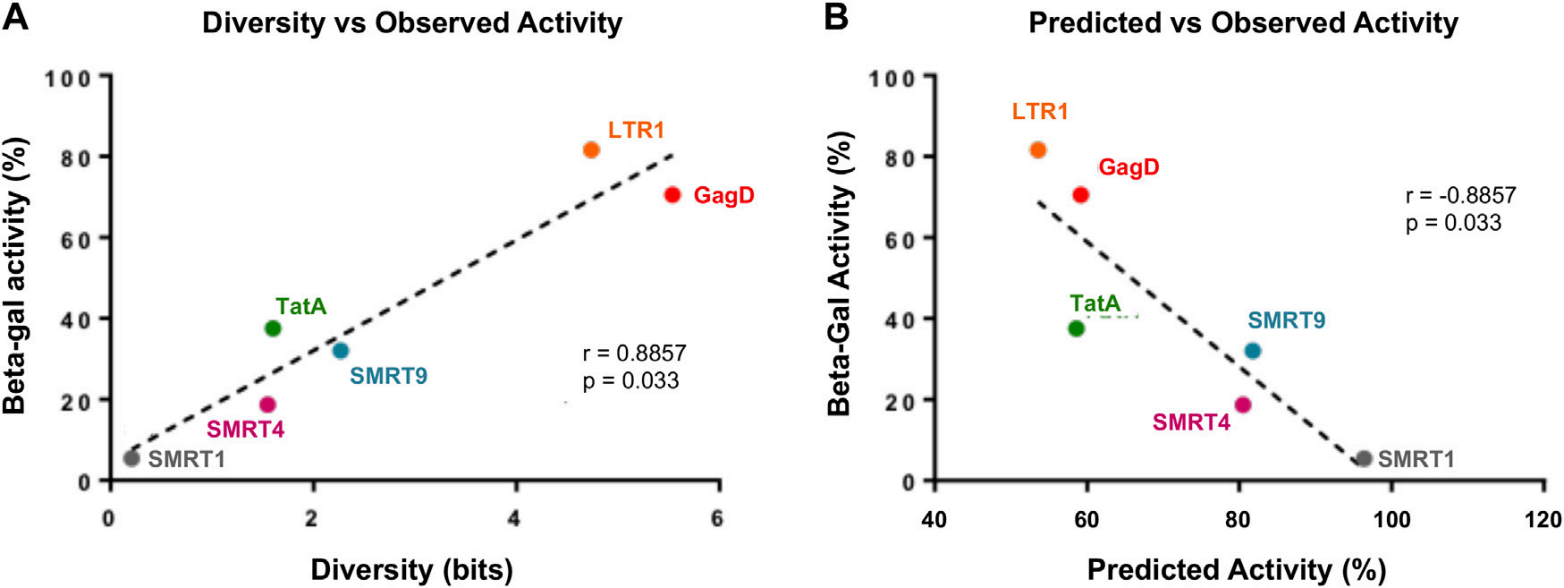
There is a strong correlation with gRNAs that target highly conserved regions and have high predicted activity profiles. Reporter expression data was taken from Figure 6. Observed activity was calculated by taking the average RLU from the three independent experiments. **(A)** Diversity was calculated for each gRNA position, details on how this was performed has been provided in the Materials and Methods. Observed activity was plotted against diversity, the dotted line represented the regression in the line. **(B)** Predicted activity was measured for each gRNA, a detailed breakdown of this calculation has been made available in the Materials and Methods. Observed activity was plotted against predicted activity, dotted line represents the regression of the line.

Finally, the data was analyzed to determine if a correlate of effectiveness could be produced with the gRNAs used in this study. The beta-galactosidase results shown in Figure 6 were used as the observed activity measure. The diversity for each target region was calculated by Shannon entropy using 424,893 HIV-1 subtype B sequences retrieved from the Los Alamos National Laboratory (LANL) database. The predicted CRISPR activity across the HIV-1 sequences in LANL was also calculated for each target region; this indicated the average cutting likelihood of the gRNA across this population of sequences. Observed activity was plotted against diversity and this showed that the more conserved a gRNA target site (score closer to 0 on *x*-axis) was the more reduction in observed activity that was acheived (lower beta-gal score) (Figure 8A). When observed activity was plotted against predicted activity the results indicated that with higher predicted activity (number closer to 1 to represent predicted cutting of 100%) the more observed activity was reduced (Figure 8B). A strong correlation was shown between both diversity and predicted activity to observed activity (Figure 8). This contrasts with what has been shown in previous reports (Wang et al., 2016; Roychoudhury et al., 2018). This was most likely due to the *in vitro* data matching with what the prediction algorithm expected. Previous reports used only bioinformatic tools, but the *in vitro* data generated did not correlate with the results generated by the algorithm.

## 4 Discussion

The purpose of this study was to determine how effective gRNAs were at inactivating HIV-1 transcription when the therapeutic package was shown to have successfully entered the cells based on the use of RFP to label a given Cas9/gRNA transcriptional product. It used gRNAs that had a perfect match to their target sequence to eliminate any mismatch penalty from these experiments, with the exception of GagD against the R7 strain. HIV-1 genetic diversity was a crucial factor when designing new gRNAs. In addition, targeting areas that are known to be vital for viral replication should be considered in the design of new gRNAs. By targeting regions of low diversity that have an important role in viral replication there is a higher chance that InDels in that region will cause adverse events during viral replication. In the present study, the effectiveness of TAR targeting gRNAs and their potential to be combined with gRNAs targeting Tat was demonstrated. While LTR-targeting gRNAs have been used previously, they have been met with varying degrees of success (Liao et al., 2015; Wang et al., 2016; Roychoudhury et al., 2018; Campbell et al., 2019; Pinto et al., 2019). One of the possible reasons for this may have been because there was limited investigation into how CRISPR was being expressed in cells that were expressing HIV-1 proteins, which is addressed in this study. Another reason could be differences in the levels of repair proteins (DNA-PK, ATM, etc.) that are expressed at different levels in different cell types. Moreover, as observed in Figure 2C even a small difference in gRNA location can have a significant effect on effectiveness. SMRT-1 and SMRT-4 are approximately 20 bps apart yet yield very different results when compared together. Taken together, this means that even if gRNAs technically target the same “region” of a gene or promoter element there can be significant differences. Most studies that have tested gRNA effectiveness against HIV-1 have done so while transfecting the CRISPR system before or while co-transfecting the HIV-1 genome. In this study, CRISPR was added before, during, and after addition of the HIV-1 genome. In concordance with most CRISPR studies when CRISPR was delivered before and at the same time as HIV-1 genome, it was the most effective. When CRISPR was administered after HIV-1 had already begun transcribing, SMRT-1 and SMRT-4 caused significant reductions in the percentage of cells that were GFP-positive, while the other LTR-targeting gRNAs did not yield a significant reduction (Figure 3). In addition, SMRT-1 was able to significantly reduce the GFP MFI, while none of the other LTR-targeting gRNAs were able to do so. This indicates that after HIV-1 transcription has been initiated it is much more difficult to stop. This is a crucial point for the CRISPR field because it highlights the importance of targeting the HIV-1 proviral genome when it is in a transcriptionally repressed or inactive stage as it is predicted to be during ART-suppression and latency. It is also not known at the time of this writing if an overabundance of substrate will overwhelm the CRISPR system when targeting the HIV-1 genome. To answer this question, co-transfections were performed at a 3:1 ratio of HIV-1 genome to CRISPR plasmid. SMRT-1 and SMRT-4 were able to significantly reduce the number of double-positive cells while the other LTR-targeting gRNAs could not (Figure 4). This data shows that even when the target substrate was administered at a higher dose, CRISPR was still able to cleave its target. In order to test LTR-targeting gRNAs against a more physiologically relevant target, a pseudotyped virus was used. The data from the pseudotyped virus infection (Figure 5) largely reflected the same trend as the prior results reported in the previous figures. SMRT-1 and SMRT-4 significantly reduced the number of double-positive cells. This data further shows that the exact position SMRT-1 targets is able to induce a significant amount of disruption to HIV-1 transcription, even when HIV-1 is integrated into the human genome. While there was not a significant difference in GFP MFI this was most likely due to a high amount of variability in the assay. The variability can likely be attributed to the elevated amount of cell death after infection with the pseudotyped virus. There have been multiple reports that have highlighted the importance of using multiple gRNAs to target the HIV-1 genome (Hu et al., 2014; Kaminski et al., 2016b; Wang et al., 2016; Bella et al., 2018; Dampier et al., 2018; Darcis et al., 2019). Because SMRT-1 is the lead candidate gRNA in this study, it was hypothesized that pairing it with a gRNA that targets Tat would have an increased effect. Figure 6 demonstrated that SMRT-1 and TatA when used together gave a 97.5 percent reduction in an LTR-driven reporter assay. It is important to note that the difference between SMRT-1 and SMRT-1 and TatA is not significant. This is likely due to the fact that SMRT-1 yielded a reduction of 95 percent, and the difference between 95 and 97.5 percent was not significant in this assay. This data also highlights the potential impact of combining a gRNA targeting the LTR and a gRNA that targets a gene coding region.

One of the most important aspects of this study is understanding how effective the CRISPR system is in a T-cell model of latent HIV-1 infection. Utilizing a latent infection model offers a unique advantage as opposed to using an acute infection to measure CRISPR effectiveness. Most likely a patient that would receive CRISPR therapy is someone who is already infected with HIV-1 and is currently adhering to ART, meaning most of the HIV-1 in the patients cells would be in an integrated latent state. The J-Lat cell line model provides a simple *in vitro* system to test the targeting capability of gRNA’s directed against an integrated provirus existing in a relatively transcriptionally repressed state. Using the J-Lat 10.6 cell line, CRISPR was successfully delivered and the data clearly indicated that a combination of SMRT-1 and TatA was a very effective combination (Figure 7). The drawback to the J-Lat system is they do not have a replication competent provirus. This means that viral replication over multiple infection cycles can not be measured. It remains an open question if the level of GFP reduced in SMRT-1- and TatA-treated cells would have given rise to replication competent virus. Although given the level of reduction observed in this assay it appears likely that replication competent virus would not be made in sufficient enough quantity to sustain multiple infection cycles.

The gRNAs designed in this study were the result of an extensive bioinformatic pipeline which was previously reported (Sullivan et al., 2019). In this report, the question that remained to be answered was whether the pipeline that was used to predict a highly active gRNA also yielded a gRNA that was indeed highly active in vitro experiments. This study clearly demonstrated that the bioinformatic pipeline was able to accurately predict the effectiveness of gRNAs (Figure 8). The correlation analysis shown in Figure 8 contrasts to what has been demonstrated in previous reports (Wang et al., 2016; Roychoudhury et al., 2018). In the Wang et al., 2016 report, the day on which breakthrough replication was observed was plotted against conservation score. This study was attempting to correlate observed activity with the level of conservation by using Shanon Entropy analysis. While the report did not show the data, it was indicated that there was not a clear correlation. In this study, it is evident that observed activity is directly correlated with conservation. This difference may be due to the effectiveness of the TAR-targeting gRNAs in this study which have a high observed and predicted activity score. The previous report did not use TAR-targeting gRNAs, but used three different LTR-targeting gRNAs. As discussed above, the variability in LTR-targeting gRNAs may have added a confounding variable to the previous analysis. The Roychoudhury et al., 2018 report offers a much more direct comparison with what was performed in this study. The previous report directly compared observed activity with predicted activity; there was a trend towards a weak positive correlation, Spearman = 0.25. In this report when comparing predicted *versus* observed activity, a Spearman correlation of −0.88 was found. The negative correlation is because in the observed activity readout, the lower the number the more effective the gRNA. Again, the difference in these data may be explained by the differences in effectiveness in the gRNAs selected for *in vitro* testing. The gRNAs used in the study by Roychoudhury et al., yielded a mean effectiveness of 15.3 percent, with a maximum effectiveness of one gRNA reaching 76.3%. In this study, the SMRT-1 gRNA consistently yielded effectiveness rates of above 90%, with multiple experiments yielding an over 95% effectiveness rate.

Multiple methods of evaluating gRNA effectiveness were used and included different HIV-1 GFP challenge times, pseudovirus infections, attempting to overwhelm the Cas9 system, sensitive biochemical readouts, and utilization of a latently infected T-cell model. While each of these systems has strengths and weaknesses, the overall trend of the data revealed that targeting highly conserved portions of the HIV-1 genome (TAR and Tat) provide the best therapeutic response. While targeting highly conserved regions of the HIV-1 genome has been important, combining that knowledge with an exacting functional rationale may lead to better gRNA selection. The SMRT9 gRNA targets a highly conserved region of the HIV-1 genome but fails to stop viral transcription in multiple assays. SMRT9 targets the beginning of the U3 region of the LTR, without a known biologic function. In contrast, SMRT1 targets the R region of the LTR, but within a region of known biological importance. These results indicate that the insertion/deletions (InDels) formed at certain locations caused functional differences in viral function, or that the virus could withstand mutations in that location. In addition, viral vectors carrying each of the gRNAs were produced and delivered successfully to a latently infected T-cell model of HIV-1 infection. Using different stimulation conditions yielded nearly identical results, indicating that even with strong stimulants that mimic T-cell activation (PMA + Ionomycin, for example,) latently infected cells were prevented from reactivating. The study also showed a correlate of effectiveness with diversity and predicted activity where used as comparators. These results have strongly solidified the rationale for using gRNAs to target highly conserved regions of the HIV-1 genome, with a known biologic function. Given the importance of TAR to the viral transcription process, there is a strong rationale for its conservation. However, future studies should compare all known gRNAs to the LTR and other regions of the genome in library screens to understand the full effect of all positions in the HIV genome that can be targeted given PAM restrictions.

Previous studies have examined a wide range of gRNAs targeting the LTR, all with varying degrees of success. There have been a small number of studies that have used gRNAs targeting the TAR region. These studies reported that those gRNAs were the most effective out of the gRNAs examined. While these previously reported gRNAs were shown to be effective, there was limited investigation into how they would account for HIV-1 genetic variation or off-target effects. We have previously used a bioinformatic pipeline to screen for gRNAs that would be able to cover the viral quasispecies found in and between patients (Sullivan et al., 2019). Moreover, the pipeline screened all gRNAs for any predicted binding to the human genome, mitigating the risk for off-target effects. However, future experiments should test the off-target effect in various models (Atkins et al., 2021b). The SMRT (SMRT1, SMRT4, SMRT9) gRNAs used in this study were generated from that pipeline. Taken together, the data presented in this study leads to the conclusion that targeting highly conserved regions of the HIV-1 genome will be imperative for any kind of therapeutic strategy. In addition, these results have demonstrated that while understanding conservation was important, combining this data with known biologic features will result in even more efficient gRNA design and execution. Moving forward utilizing a similar approach to design a highly effective gRNA against Tat that is able to cover as much of the patient quasispecies as possible while minimizing off-target effects should be performed. Once accomplished, combining SMRT1 and a next-generation Tat gRNA could be a very effective combination to stop HIV-1 gene expression.

## Data Availability

The original contributions presented in the study are included in the article/Supplementary Material, further inquiries can be directed to the corresponding author.

## Supplementary material

Supplementary Table S1 Figures relating to supplemental data.
