# Supplementary material for: Assessment of anti-HIV-1 guide RNA efficacy in cells containing the viral target sequence, corresponding gRNA, and CRISPR/Cas9
Source: Front Genome Ed. 2023 Apr 13;5:1101483. doi: 10.3389/fgeed.2023.1101483 (PMC10134072; doi:10.3389/fgeed.2023.1101483)
Supplement: Supplementary file 1 [file DataSheet1.docx]

Supplementary Material

# Supplementary Data

**Figure S1.** gRNAs targeting the TAR regions have a decreased amount of double-positive cells by fluorescent microscopy.

**Figure S2.** S1 and S4 were able to significantly reduce the amount of HIV-1 GFP expression when exposed to three times the amount of HIV-1 GFP DNA.

**Figure S3.** A 10:1 ratio of S1 to HIV-1 GFP was able to show significant reduction while maintaining reporter gene expression.

**Figure S1. gRNAs targeting the TAR regions have a decreased amount of double-positive cells by florescent microscopy.** HEK-293T cells were transfected as described in Figure 2. Pictures were taken using an Olympus IX81 microscope at a 10X objective 48 hours post transfection with HIV-1 GFP. The TRITC channel was used to detect expression of RFP and the FITC channel was used to detect GFP. The merge channel is an overlay of BF, TRITC, and FITC. BF = brightfield

**Figure S2. S1 and S4 were able to significantly reduce the amount of HIV-1 GFP expression when exposed to three times the amount of HIV-1 GFP DNA.** HEK-293T cells were transfected as described in Figure 4. Forty eight hours post transfection cells were imaged on an Olympus IX81 microscope. The TRITC channel was used to detect expression of RFP and the FITC channel was used to detect GFP. The merge channel is an overlay of BF, TRITC, and FITC. BF = brightfield

**Figure S3. A 10:1 ratio of S1 to HIV-1 GFP was able to show significant reduction while maintaining reporter gene expression.** TZM-bl cells were seeded in 96-well plates at 20,000 cells per well. Cells were then co-transfected with HIV-1 GFP and Cas9 plasmids. Forty eight hours post transfection, cells were processed for beta-galactosidase expression. Relative light units (RLU) was used to determine the amount of beta-galactosidase produced.
